# Anti-amyloid therapies do not slow Alzheimer's disease progression

**DOI:** 10.1590/1980-5764-DN-2023-0099

**Published:** 2024-01-05

**Authors:** Markku Kurkinen

**Affiliations:** 1 NeuroActiva, Inc., San Jose CA, United States of America.

Dear Editor,

In his letter “Anti-amyloid therapies work for Alzheimer's disease” to the Editor of Brain Communications, Sir John Hardy voices his opinion: “Anti-amyloid therapies slow Alzheimer's disease progression… the argument about whether these agents slow disease is now settled”^1^. Here, following his previous comments^2^, Hardy is referring to the outcomes of recent clinical trials of lecanemab^3^ and donanemab^4^, two anti-Aβ monoclonal antibodies approved by the Food and Drug Administration (FDA) for the treatment of Alzheimer's disease (AD) patients. However, even a casual look at these trial reports reveals misinterpretation of the data and trivial miscalculation of the antibodies’ clinical benefit^5–7^.

Clinical Dementia Rating-Sum of Boxes (CDR-SB) measures cognition and function on an 18-point scale, higher scores indicating worse impairment. CDR-SB consists of six tests, each scored 0, 0.5, 1, 2 or 3. In the lecanemab trial reported by van Dyck et al.^3^, the CDR-SB score at the trial end was 4.41 with lecanemab, a change of 1.21 from the 3.2 baseline, and 4.86 with placebo, a change of 1.66. The difference of 0.45 of the score changes between the lecanemab and placebo treated trial participants is below 0.5, the minimum observable measure on CDR-SB scale. The 0.45 difference has been interpreted as 27% (0.45/1.66) slowing of disease progression and cognitive decline with lecanemab compared to placebo. However, it's not the score changes but the 4.41 and 4.86 scores for cognition and function that matter, and these are the scores that must be used when calculating lecanemab's clinical benefit. Accordingly, lecanemab treated study participants have 9.3% (0.45/4.86) less cognitive and functional impairment compared to placebo^5,6^. Further, lecanemab did not work for women, which is bad news because there are two times more women than men with AD. Lecanemab did not work for APOE4 carriers, which is bad news for AD patients, 60–75% of whom have one or two APOE4 gene^6^. Remarkably, for some reason, these observations were not explicitly stated, not even discussed, in the report by van Dyck et al.^3^, only to be found in the Supplementary Appendix (Fig. S1B), and thus have been absent in the commentaries, public discussion and news media.

In the donanemab trial^4^, CDR-SB score was 4.64 in the donanemab group, a change of 1.20 from baseline, and 5.13 in the placebo group, a change of 1.88. The difference of 0.68 of the score changes has been interpreted as 36% (0.68/1.88) slowing of cognitive decline. However, 4.64 and 5.13 (difference 0.49) are the scores that must be used when calculating the effect of donanemab treatment compared to placebo. Accordingly, 9.6% (0.49/5.13) less impairment is a better estimate for donanemab's clinical benefit^7^. When cognition and function were measured on iADRS, a 144-point scale, lower scores indicating worse impairment, the score was 101.31 in the donanemab group, a change of 6.02 from baseline, and 98.88 in the placebo group, a change of 9.27. The difference of 3.25 of the score changes has been interpreted as 35.1% (3.25/9.27) slowing of decline. However, 101.31 and 98.88 (difference 2.43) are the iADRS scores that matter in the end; therefore, 2.5% (2.43/98.88) less impairment is donanemab's clinical benefit^7^.

These small score changes, trending in favor of lecanemab and donanemab, were observed in randomized, double-blind, placebo controlled clinical trials in early AD patients. In these trials, placebo was saline-solution, which is not an adequate control for anti-amyloid antibodies. Certainly, immune system reacts differently to antibody proteins compared to saline solution. To what extend this ‘placebo-effect’ can obscure results in anti-amyloid therapies has not been studied.

Hardy ends his letter: “…we should not waste our time arguing about whether amyloid has been a legitimate or successful disease target. Clearly, it was”. And I could not agree more; except, clearly it was not.
